# Software algorithm and hardware design for real-time implementation of new spectral estimator

**DOI:** 10.1186/1475-925X-13-61

**Published:** 2014-05-09

**Authors:** Edward J Ciaccio, Angelo B Biviano, Hasan Garan

**Affiliations:** 1 Department of Medicine–Division of Cardiology, Columbia University Medical Center, New York, USA; 2 Columbia University, Presbyterian Hospital 7 W-318, 630 West 168th Street, New York NY 10032, USA

**Keywords:** Algorithm, Analog computer, Circuit design, Digital computer, Spectral analyzer

## Abstract

**Background:**

Real-time spectral analyzers can be difficult to implement for PC computer-based systems because of the potential for high computational cost, and algorithm complexity. In this work a new spectral estimator (NSE) is developed for real-time analysis, and compared with the discrete Fourier transform (DFT).

**Method:**

Clinical data in the form of 216 fractionated atrial electrogram sequences were used as inputs. The sample rate for acquisition was 977 Hz, or approximately 1 millisecond between digital samples. Real-time NSE power spectra were generated for 16,384 consecutive data points. The same data sequences were used for spectral calculation using a radix-2 implementation of the DFT. The NSE algorithm was also developed for implementation as a real-time spectral analyzer electronic circuit board.

**Results:**

The average interval for a single real-time spectral calculation in software was 3.29 μs for NSE versus 504.5 μs for DFT. Thus for real-time spectral analysis, the NSE algorithm is approximately 150× faster than the DFT. Over a 1 millisecond sampling period, the NSE algorithm had the capability to spectrally analyze a maximum of 303 data channels, while the DFT algorithm could only analyze a single channel. Moreover, for the 8 second sequences, the NSE spectral resolution in the 3-12 Hz range was 0.037 Hz while the DFT spectral resolution was only 0.122 Hz. The NSE was also found to be implementable as a standalone spectral analyzer board using approximately 26 integrated circuits at a cost of approximately $500. The software files used for analysis are included as a supplement, please see the Additional files 1 and 2.

**Conclusions:**

The NSE real-time algorithm has low computational cost and complexity, and is implementable in both software and hardware for 1 millisecond updates of multichannel spectra. The algorithm may be helpful to guide radiofrequency catheter ablation in real time.

## Introduction

The discrete Fourier transform (DFT) is used ubiquitously for power spectral estimation of biomedical data. However, it does not readily lend itself to real-time analysis. When successive snapshots of the signal spectrum are desired [1], a real-time spectral estimator is needed. Early work found that it was possible to implement recursive and nonrecursive procedures to update the DFT estimate when an analysis window of N values (i.e., an ordered set, or block) is staggered by a moving size of M sample points, with both N and M being powers of two [1]. Using a complex split-radix implementation, it is possible to improve efficiency by 2-3 × for real-time update as compared with the radix-2 implementation [2]. Other algorithms have been developed that specifically target the implementation of the two-dimensional DFT, with reduction of up to 50% in the computation time being observed [3]. Similarly, a split-vector-radix implementation for two-dimensional DFT analysis was found to reduce the computation time to half or less as compared to the MATLAB implementation [4]. However, this latter algorithm works best for real-time analysis only when the moving size M is large, i.e., there is the need to skip samples, or when the analysis window N is shortened, in order for real-time DFT update to be feasible. Yet increasing M or decreasing N will result, respectively, in a diminished time and frequency resolution of the real-time analysis. Ideally, a sliding window moving size of M = 1 sample point, (i.e., spectral update for every new input sample point) should be used so that the real-time spectral analysis can capture all frequency changes. The optimal window length for atrial fibrillation (AF) frequency analysis was shown to be N = 8192 sample points in a prior study [5].

The development of a real-time spectral analyzer having moving size M = 1, window length N = 8192 discrete sample points, and low computational overhead would potentially be useful for mapping of AF electrograms in order to detect and localize arrhythmogenic regions for radiofrequency ablation. Although current frequency analysis methods hold some promise for this purpose [6,7], due to a lack of resolution, the algorithms are unable to capture transient periodicity, i.e., periodic signal components that are present for only brief intervals, which are likely related to independent, intermittent drivers of the arrhythmia. If an algorithm for real-time spectral update were to be devised without reducing the time or frequency resolution, it could be helpful to detect transient electrophysiologic events, as well as to improve analysis of the detail and trends in sequentially-acquired atrial electrical activation data. In this study both a real-time software algorithm and a hardware design (prototypical hardware electronics board block diagram) for power spectral analysis are described based on a new spectral estimator (NSE) [8-11] which is applied to fractionated atrial electrogram data.

## Method

### Clinical data acquisition

Atrial electrograms were recorded from patients referred to the Columbia University Medical Center cardiac electrophysiology laboratory for catheter ablation of the AF substrate. These recordings were obtained prospectively as approved by the Institutional Review Board, and analyzed retrospectively for this study. Nine patients had documented clinical paroxysmal AF. Ten other patients had longstanding persistent AF that did not terminate for several months or years prior to catheter mapping and ablation. The atrial mapping procedure was done using a NaviStar ThermoCool catheter, 7.5 F, with 3.5 mm tip and a 2 mm spacing between the bipoles of the distal ablation electrode (Biosense-Webster Inc, Diamond Bar, CA, USA). The electrogram signals were acquired using a General Electric CardioLab system (GE Healthcare, Waukesha, WI), and filtered at acquisition from 30 to 500 Hz with a bandpass filter (single-pole) to remove baseline drift and high frequency noise. The filtered signals were then sampled at 977 Hz (i.e., approximately 1 millisecond intervals between samples) and the digital data was stored to PC-type computer disk. Although the low pass corner frequency was slightly above the Nyquist value, negligible electrophysiologic signal energy resides above this range [9]. The data was extracted without patient identifiers to a USB drive for subsequent analysis. As in previous studies, to standardize the morphological characteristics, all CFAE were preprocessed to mean zero and unity variance (average signal level = 0 volts, standard deviation and variance = 1).

### The NSE algorithm

For computer implementation of the NSE, a software algorithm was developed. The NSE power spectrum is formed from the ensemble averages of signal segments. When successive signal segments of length w are added together, if the segments are correlated, then the sum will reinforce the individual components, which would indicate periodicity at an interval w. The ensemble mean vector e_w_ of dimension w × 1 is defined as the average of n successive segments of an N × 1 dimensional signal ${\underset{-}{x}}_{N}$, where ${\underset{-}{x}}_{N}$ is normalized to mean zero and unity variance prior to analysis. Each segment ${\underset{-}{x}}_{w,i}$ of this signal, of dimension w × 1, is used for averaging:

(1) $${\underset{-}{e}}_{w}=\frac{1}{n}\sum_{i}{\underset{-}{x}}_{w,\;i}\;i=1\;\mathrm{to}\;n$$

where underscore indicates a vector quantity, the first subscript denotes the vector length, and:

(2) $${\underset{-}{x}}_{N}=\left[\begin{array}{c} {\underset{-}{x}}_{w,1}\\ {\underset{-}{x}}_{w,2}\\ \ldots \\ {\underset{-}{x}}_{w,n} \end{array}\right]$$

with *x*_*w,i*_ being signal segments of length w, from i = 1 to n, with the number of summations for averaging, n, given by:

(3) $$n=\mathrm{int}\left(\frac{N}{w}\right)$$

and *int* is the integer function (decimal is rounded down). For each ensemble mean vector, the sum of squares of all elements divided by the vector length is defined as the ensemble power. This can be expressed as:

(4) $$P_{w}=\frac{1}{w}\cdot {\underset{-}{e}}_{w}^{T}\cdot {\underset{-}{e}}_{w}$$

The NSE power spectrum is plotted as:

(5) $$\sqrt{n}\;P_{\mathrm{wRMS}}=\sqrt{n}\;\sqrt{P_{w}}$$

where P_wRMS_ is the root mean square power, and the scaling term √n is used to counter the falloff of the baseline level by 1/√n per number of summations n used for signal averaging [9]. From Eq.’s 1 and 4:

(6a) $$P_{w}=\frac{1}{w}\cdot \left(\frac{1}{n}\sum_{i}{\underset{-}{x}}_{w,\;i}\;\cdot \frac{1}{n}\sum_{i}{\underset{-}{x}}_{w,\;i}\right)$$

(6b) $$\;=\frac{1}{n^{2}w}\cdot \left(\sum_{i}{\underset{-}{x}}_{w,\;i}\;\cdot \sum_{i}{\underset{-}{x}}_{w,\;i}\right)$$

where *i* is the segment number from 1 to n. Thus from Eq.’s 5 and 6a:

(7a) $$\sqrt{n}\;P_{\mathrm{wRMS}}=\sqrt{\frac{n}{n^{2}w}\cdot \left(\sum_{i}{\underset{-}{x}}_{w,\;i}\;\cdot \sum_{i}{\underset{-}{x}}_{w,\;i}\right)}$$

(7b) $$=\sqrt{\frac{1}{N}\cdot \left(\sum_{i}{\underset{-}{x}}_{w,\;i}\;\cdot \sum_{i}{\underset{-}{x}}_{w,\;i}\right)}$$

As in prior studies [8-11], Eq. 7b was used to implement the offline version of the NSE power spectral calculation.

The real-time NSE power spectral calculation was developed so as to estimate the ensemble mean from a moving average. By using a moving average, only one calculation is needed to update e_w_ for each power spectral calculation, rather than n calculations as in Eq. 1. From Eq.’s 4 and 5, the normalized root mean square power is:

(8a) $$\sqrt{n}\;P_{\mathrm{wRMS}}=\sqrt{n}\;\sqrt{\frac{1}{w}\cdot {\underset{-}{e}}_{w}^{T}\cdot {\underset{-}{e}}_{w}}$$

(8b) $$=\sqrt{\frac{n}{w}}\;\sqrt{{\underset{-}{e}}_{w}^{T}\cdot {\underset{-}{e}}_{w}}$$

(8c) $$=\frac{\sqrt{N}}{w}\;\sqrt{{\underset{-}{e}}_{w}^{T}\cdot {\underset{-}{e}}_{w}}$$

(8d) $$=\frac{n}{\sqrt{N}}\;\sqrt{{\underset{-}{e}}_{w}^{T}\cdot {\underset{-}{e}}_{w}}$$

where the √N term is a constant, and for the N = 8,192 sample point window size used in this study, it has a value of 90.51, while 1/√N = 0.01105. For real-time power spectral updates, a moving average < e_w_ > is substituted for e_w_ in Eq. 8d, thus:

(9) $$\sqrt{n}\;P_{\mathrm{wRMS}}=\frac{n}{\sqrt{N}}\;\sqrt{{\langle {\underset{-}{e}}_{w}\rangle }^{T}\cdot \langle {\underset{-}{e}}_{w}\rangle }$$

For both the offline and real-time NSE power spectral calculation, √n P_wRMS_ is plotted versus frequency f, which is given by:

(10) $$f=\frac{\mathrm{sample}\_\mathrm{rate}}{w}$$

The ensemble power is computed in the standard electrophysiologic frequency range of 3-12 Hz [12,13]. At the 977 Hz sampling rate, this corresponds to a range of w from 325-81 discrete sample points. Thus in the electrophysiologic frequency range, the NSE is a 245 point power spectrum. The signal length N for analysis does not affect the spectral resolution, but it does affect the number of summations for averaging used to calculate e_w_ and P_w_. Greater number of summations improves cancellation of random components while forming the ensemble mean; however, the DF may not be stable over long intervals [14,15]. Over short intervals, the NSE estimate will be improved when the signal is composed mostly of periodic components and less random noise. Although the DFT does not depend on a summation of signal components, the DFT frequency resolution is inversely proportional to the window length; thus short intervals have low resolution.

### Software implementation

A computer algorithm to implement the offline NSE power spectrum calculation is shown below (called spectral_estimate). In the source code, period w ranges from n1 = 325 to n0 = 81 sample points (3-12 Hz). The calculation window is n2 = 8,192 sample points, which is approximately 8 seconds when sampled at a rate r = 977 Hz. The number of signals (multichannels) analyzed n3 = 216. For calculation, the input data array, with each patient data being received from a separate file, is ‘inp’ (lines 4-6), the ensemble mean is stored in array ‘en’ (lines 7-15), and the generated spectrum is stored in array ‘s’ (lines 16-19). Note that line 17 in the software code arises from Eq. 7b. For simplicity, the spectrum of a single electrogram was written to disk, arbitrarily taken as channel 4 (line 18). For faster run time, lines 8-12 calculate the ensemble means from w = 163 to w = 325. Then in lines 13-15, the ensemble means from w = 162 to w = 81 are computed by simply adding the two halves of vector 2w that is calculated by lines 8-12, thus:

(11) $$\left[\begin{array}{c} e_{w,1}\\ e_{w,2}\\ \ldots \\ e_{w,w} \end{array}\right]=\left[\begin{array}{c} e_{2w,1}\\ e_{2w,2}\\ \ldots \\ e_{2w,w} \end{array}\right]+\left[\begin{array}{c} e_{2w,w+1}\\ e_{2w,w+2}\\ \ldots \\ e_{2w,2w} \end{array}\right]$$

is determined in lines 13-15, which reduces redundancy in calculation. The program spectral_estimate is used for offline (non real-time) computations, i.e., it calculates a single spectrum for each patient record using sample points 1-8192, and it has been used in prior studies [8-11]. The offline time for computation of a single NSE spectrum for 216 patient records was compared with the offline radix-2 implementation of the Fast Fourier Transform (FFT) [16]. The time for spectral estimation, lines 7-20 was determined using the ‘date_and_time’ Fortran function, which was inserted between lines 6-7 and between lines 20-21 in the actual source code. Using the same ‘date_and_time’ function, the run time for spectral estimation using the offline radix-2 FFT implementation was also determined. Since slight temporal changes in processor speed can occur, the mean and standard deviation in runtime over five trials was calculated for both the NSE and FFT methods.

**Figure 1:**
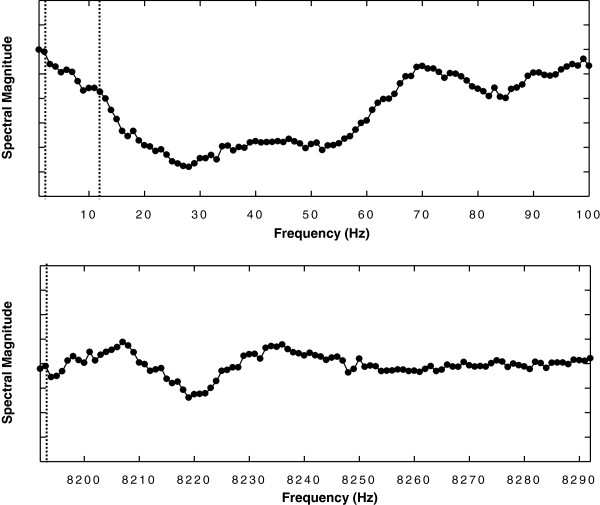
**Windows used for analysis.** A moving average was used, which was calculated based upon prior discrete sample points in the sequence.

The NSE and FFT spectral estimators were then implemented for real-time analysis. An update was computed once every sample point (moving size M = 1). In Figure 1, a fractionated electrogram is shown. Calculations of the ensemble mean were done using a moving average. Early in the sequence, for example at discrete sample points 2 and 12 (show as dashed vertical lines in the top graph), the moving average was based upon the few available prior data points and was a rough estimate. Later in the sequence, for example at sample point 8192 (dashed vertical line in lower graph), the moving average was based upon many prior data points. The software code for real-time NSE analysis is shown following (spectral_estimate_real_time). The declaration lines are virtually the same as for the offline program (spectral_estimate). In the real-time program, most variables are vectors whose elements represent a range in w from 81 to 325. Declaring length w as an integer was found to considerably reduce computation time as compared to a floating point declaration. Lines 5-8 compute constants c1 and c2 for a moving average, or low pass filter, which is used iteratively for a subsequent calculation in the coding sequence. Lines 9-11 input the data, the same as lines 4-6 in the offline program (spectral_estimate). However the ensemble mean calculation (lines 12-18) is slightly different as compared with the spectral_estimate program. The actual calculations are done in lines 15-17. The index ‘ind’ is first calculated (line 15), which is used to point to a particular element within each vector that is used for calculation. The index is incremented by 1, and after it reaches a value of w, it wraps around to a value of 1. On line 15 the prior value of the ensemble mean at the index is also stored in a buffer (ee). The ensemble mean is then updated using the moving average (line 16), which includes the constants c1 and c2 calculated in lines 5-8. The real-time ensemble mean calculation can be expressed as:

(12) $${\underset{-}{e}}_{w,\;i}=c1\cdot {\underset{-}{e}}_{w,\;i-1}+c2\cdot {\underset{-}{x}}_{w,\;i}$$

where:

(13) $$c1=\frac{n-1}{n}$$

(14) $$c2=\frac{1}{n}$$

with n, and therefore c1 and c2, being dependent upon the value of w (Eq. 3).

The power of the ensemble mean vector of length w is then updated in line 17. The ensemble power can be expressed as:

(15) $$P_{w}=e_{1}\cdot e_{1}+e_{2}\cdot e_{2}+\ldots +e_{w}\cdot e_{w}$$

where e is the scalar ensemble mean, index *i* ranges from 1 to w, and the divide by w is accounted for when the spectral point is calculated subsequently in the code. At any particular index value *i* = 1 to w, the prior squared ensemble mean *e*_*i* − *w*_ · *e*_*i* − *w*_, which was calculated w sample points previously in time, is removed, and it is replaced with the newly calculated value *e*_*i*_ · *e*_*i*_. Thus at each index *i*, the power is updated as:

(16) $$P_{w}=P_{w}-e_{i-w}\cdot e_{i-w}+e_{i}\cdot e_{i}$$

where the index i is incremented by 1 with each new input sample point. For each new sample point, all *P*_*w*_ are updated using Eq. 16. All spectral points *S*_*w*_ are then calculated as the square roots of *P*_*w*_, scaled by n(w)/√N as in Eq. 8d, with 1/√N symbolized by xn in lines 1 and 17 of the spectral_estimate_real_time software program. It was found that implementation using Eq. 8d rather than 8c, so that there is no actual ‘divide by’ in the computation, considerably reduces the runtime. One complete spectrum, arbitrarily selected as that for data record 4, is stored for subsequent analysis (lines 19-22). The frequency variable, which is the x-axis parameter for graphing, is also stored (r / w, line 21).

Note in the spectral_estimate_real_time program that 16,384 spectra are calculated for each of 216 patient records (lines 12-13). This program is designed as a real-time implementation, updating the spectrum with each new input sample point. However, it will only be justified as being an actual real-time algorithm if the update calculations for all channels can be done at a speed faster than the discrete sampling interval, which is approximately 1 millisecond. As for the spectral_estimate program, the time for spectral estimation using spectral_estimate_real_time was determined by inserting the ‘date_and_time’ Fortran library function at the appropriate locations in the code (between lines 11 and 12, and after line 23).

The radix-2 FFT program was also implemented for real time [16]. For ease of comparison, all FFT calculations were repeated for each sliding analysis window rather than using any of the shortcuts suggested by other authors [1,2]. Because the repetition of FFT calculations is time-consuming, it was run for only 512 input sample points. Again inserting the Fortran ‘date_and_time’ function at appropriate locations in the coding sequence, the time for FFT calculation over 512 sliding windows was determined, which was then scaled by 32 × for comparison with the NSE implementation in which spectra were calculated for 16,384 sliding windows. Since slight temporal changes in processor speed can occur, the mean and standard deviation in spectral computation time over five trials was determined for both the NSE and DFT real-time methods.

As an additional test of the real-time NSE implementation, spectral parameter values at sample point 8,192 were computed for the 216 data files, and the results were compared to values computed using the offline NSE implementation. It was shown previously that significant differences exist in the dominant amplitude (DA), dominant frequency (DF), and mean and standard deviation in spectral profile (MP and SP, respectively) of paroxysmal versus persistent AF fractionated electrogram recordings when using the NSE and DFT offline implementations [17]. The DA is defined as the amplitude and the DF is the frequency of the largest fundamental peak in the power spectrum over the physiologic range of 3-12 Hz [17]. The MP and SP are, respectively, the mean and standard deviation of the spectral profile after the magnitude has been normalized to a range between 0 and 1. Unlike the regularity and organizational indices, spectral parameters which require guestimates of the width of the DF spectral peak and of its harmonics [18,19], no such guestimates are required to calculate the DA, MP, and SP parameters. These new parameters have also been incorporated in the study of another group [20]. If real-time implementation could produce similar means and standard deviations in these spectral parameters, and similar significant differences, it would provide evidence that the real-time NSE implementation is similar to the offline implementation.

The NSE and FFT source code was compiled using an Intel Visual Fortran Compiler (Standard Edition for Windows, Version 9, Intel Corporation, Santa Clara CA, 2005). All computer calculations were done using a PC-type laptop having the following specifications:

Model: Lenovo X201

Operating system: Windows XP Professional

Version: 2002, Service pack 3 (32 bit)

Central Processing Unit (CPU): Intel Core i5 M 540 @ 2.53 GHz

Random Access Memory (RAM): 2.92 Gbytes

The software code and data files used for analysis in this study are included as a supplement (please see Additional files 1 and 2).

### Hardware implementation

Lines 13-23 of the spectral_estimate_real_time program were then implemented in schematic form in hardware. For ease of implementation, CMOS process integrated circuits were selected to do the calculations shown in the software code. The integrated circuits were also selected for their utility and high performance. The goal was to implement the calculations on a small prototype electronics board. A requirement for implementation was that the electronics board would operate in standalone mode, without any computer control. For simplicity, the delays needed to ensure system stability and proper handshaking were not shown, but can be readily implemented with a 555 timer circuit to provide a delay for latching data. An approximate cost for parts was determined based on the schematic hardware implementation.

## Results

### Overview

The DFT spectral resolution in this study is given by:

(17) $$\mathrm{SR}=\frac{\mathrm{rate}}{\mathrm{window}\;\mathrm{length}}=\frac{1\;\mathrm{kHz}}{8192\;\mathrm{samples}}=0.122\mathrm{Hz}$$

Thus in the range 3-12 Hz, the DFT spectrum will consist of 9/0.122 ≈ 74 spectral points. The mean NSE spectral resolution for this study is approximated by [11]:

(18) $$\mathrm{SR}\approx \frac{1}{245}\sum_{w}\frac{\mathrm{rate}}{w^{2}}\approx \frac{1\;\mathrm{kHz}}{245}\sum_{w}\frac{1}{w^{2}}\;\mathrm{for}\;w=325\;\mathrm{to}\;81$$

**Figure 2:**
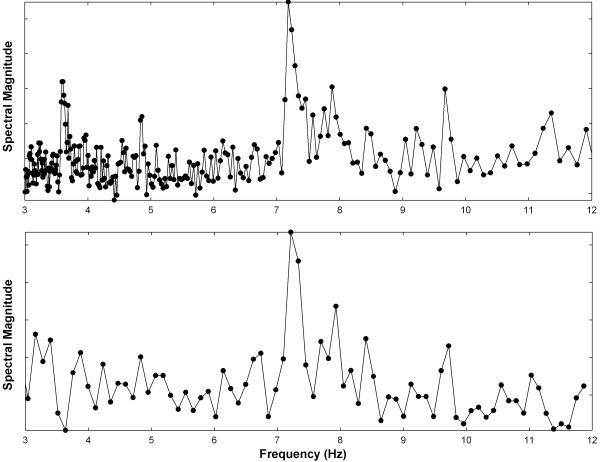
Comparison of offline NSE (top) versus DFT (bottom) power spectra for a signal acquired from the right inferior pulmonary vein antrum in a longstanding persistent AF patient.

The NSE spectrum in the range 3-12 Hz consists of 245 spectral points, and from Eq. 18, the mean resolution is 0.037 Hz. The NSE spectral resolution is therefore improved by more than 3 × over the DFT spectral resolution for this study.Examples of power spectra for the offline NSE and DFT implementations are depicted in Figure 2. The spectra were generated from a sequence acquired from the antrum of the right inferior pulmonary vein in a persistent AF patient using the distal ablation catheter bipolar electrode. The DF occurs at approximately 7.2 Hz, and a secondary peak, perhaps caused by an independent generator of electrical activity in the left atrium, is present at approximately 9.7 Hz. The individual spectral points are shown by solid circles. The DFT resolution is fixed at 0.122 Hz and consists of approximately 74 spectral points. The NSE resolution varies with frequency and consists of 245 spectral points. This makes the NSE spectral peaks better defined and sharper.

**Figure 3:**
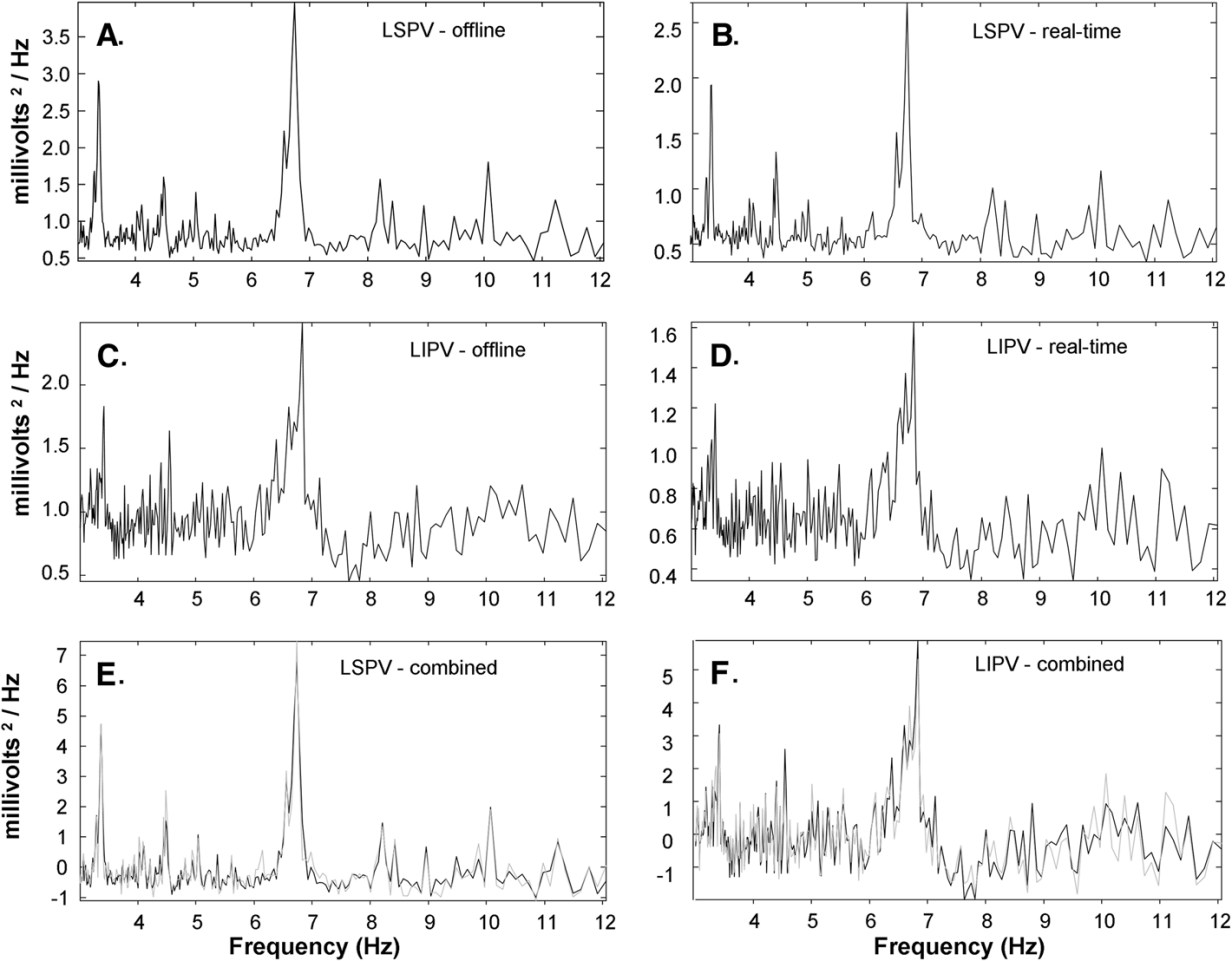
**Spectral estimates using the offline (panels A and C) and real-time (panels B and D) NSE software algorithm.** Data used for analysis was acquired from the same patient at the antrum of the pulmonary veins–left superior **(panels A and B)** and left inferior **(panels C and D)**. Overlap between offline and real-time traces is shown in **panels E and F**.

Graphs of real-time NSE spectral estimates were constructed for a patient with longstanding persistent AF. The spectral estimates were generated from fractionated electrogram records. In Figure 3, instances are shown of the spectral estimate using the original NSE algorithm spectral_estimate (panels A and C) and the real-time NSE algorithm spectral_estimate_real_time (panels B and D). Panels A and B show a recording from the left superior pulmonary vein antrum in a patient with persistent AF, and panels C and D were recorded from the left inferior pulmonary vein antrum in the same patient. For spectral_estimate, the result of analysis of the static window with data points 1-8,192 is shown. For spectral_estimate_real_time, the result of analysis at sample point 8,192 is shown (since the analysis windows are moving averages). There are only subtle differences in the result as can be seen in panels E and F, where the spectra are overlapped after scaling to mean zero and unity variance (offline–black, real-time–gray). The root mean square error difference is 0.23 ± 0.21 mV^2^ in panel E and 0.34 ± 0.26 mV^2^ in panel F.

**Figure 4:**
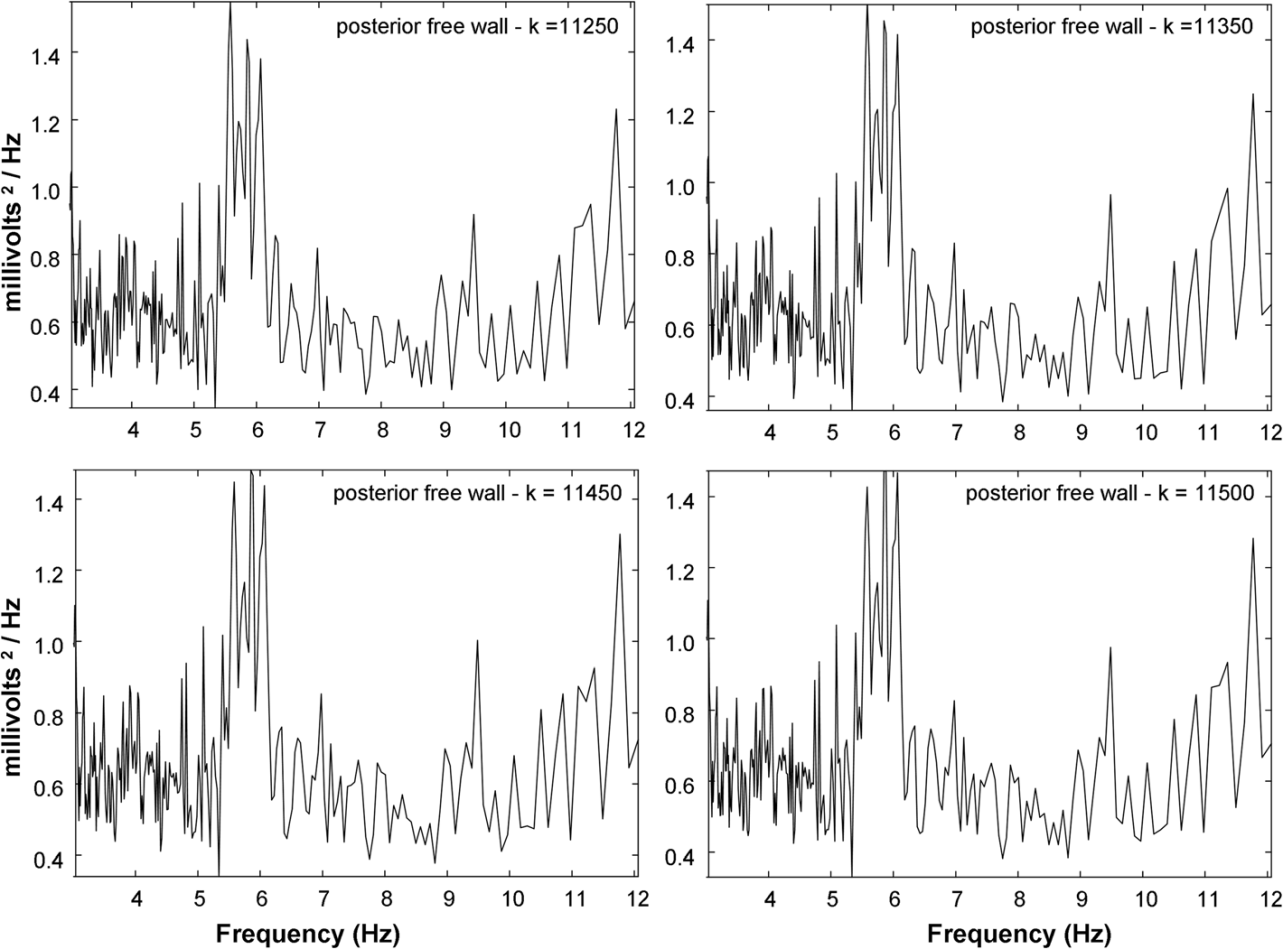
**NSE spectra using the real-time software algorithm for small shifts of 50-100 milliseconds in analysis window location.** Changes in the tallest of the split peaks can be observed over time.

**Figure 5:**
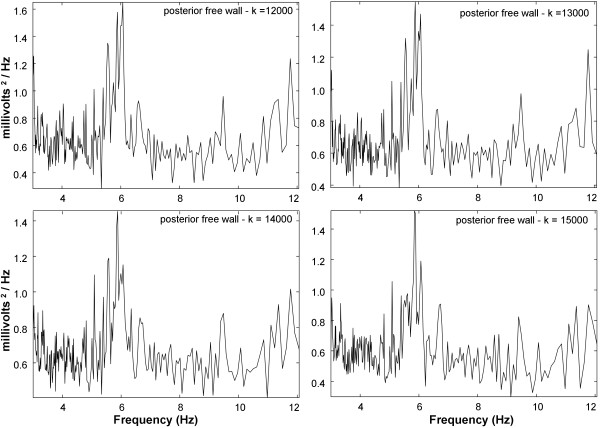
**NSE spectra using the real-time software algorithm for larger change in analysis window location of 1000 milliseconds.** Changes from split peak to single peak can be observed over time.

The statistical results for paroxysmal versus persistent AF spectral parameters are shown in Table 1. The means and standard deviations for spectral parameters in persistent versus paroxysmal AF patients are noted. For both the offline and real-time algorithms, the DA parameter is of greater magnitude in persistents versus paroxysmals and the difference is highly significant (p < 0.001). Similarly, for both offline and real-time algorithm, the DF parameter is higher in persistents versus paroxysmals and the difference is highly significant (p < 0.001). Furthermore, for both offline and real-time algorithm, the MP and SP parameters are of lesser magnitude in persistents versus paroxysmals and the difference is highly significant (p < 0.001). The measurements suggest that electrical activity is less organized in paroxysmal AF as compared with persistent AF [17], though ablation of high DF sites has been shown to more likely result in arrhythmia termination for paroxysmal AF [7]. The means and standard deviations of all parameters have similar values for the offline versus real-time algorithm (Table 1). There are no significant differences in the means of offline versus real-time parameters for persistent AF (P ≥ 0.201) and for paroxysmal AF (P ≥ 0.302). Therefore, the real-time implementation provides a similar spectral estimate to the offline implementation at a particular time epoch.To illustrate how the time-varying spectral content can be detected using the real-time algorithm, Figures 4 and 5 show spectra from successive points in time for data acquired at the posterior left atrial free wall in a patient with persistent AF. The split peaks at the DF change slightly over short time intervals of 50-100 milliseconds between frames as in Figure 4. Over longer intervals of 1000 milliseconds, major changes in the spectra occur (Figure 5). The split peaks at the DF (k = 12,000 and 13,000 sample points) become single peaks (k = 14,000 and 15,000 sample points). Over successive frames in Figure 5, the background level becomes somewhat reduced in magnitude, particularly at lower frequencies for the spectra acquired at k = 15,000 sample points. Thus temporal changes in spectral details are evident over both short and longer intervals using the real-time implementation of NSE.

**Table 1:** Statistics

**Type**	**Parameter**	**Persistent**	**Paroxysmal**	**Significance**
Offline	DA	1.848 ± 0.602	1.478 ± 0.301	P < 0.001
Offline	DF	6.254 ± 1.004	5.378 ± 1.167	P < 0.001
Offline	MP	0.342 ± 0.105	0.404 ± 0.078	P < 0.001
Offline	SP	0.156 ± 0.024	0.168 ± 0.019	P < 0.001
Real-time	DA	1.848 ± 0.603	1.484 ± 0.304	P < 0.001
Real-time	DF	6.253 ± 0.900	5.598 ± 1.106	P < 0.001
Real-time	MP	0.329 ± 0.105	0.399 ± 0.079	P < 0.001
Real-time	SP	0.159 ± 0.025	0.170 ± 0.021	P < 0.001
Off vs Real	DA	P = 1.000	P = 0.837	–
Off vs Real	DF	P = 0.946	P = 0.302	–
Off vs Real	MP	P = 0.462	P = 0.883	–
Off vs Real	SP	P = 0.201	P = 0.370	–

Off vs Real = Offline versus Real-time, DA = dominant amplitude, DF = dominant frequency, MP = mean spectral profile, SP = standard deviation in spectral profile.

### Summary software statistics

Computation of single (i.e., offline) 8,192-point spectra for 216 patient records completed in 0.38 ± 0.01 seconds using NSE and in 0.12 ± 0.01 seconds using the radix-2 FFT implementation. Thus the offline FFT calculation is more than 3 × faster than the NSE. This result can be explained by the increased number of calculations required by NSE for a single spectral estimate as compared to FFT. For real-time analysis, 16,384 spectra from 216 patient records (3,538,944 spectra total) were calculated in 11.63 ± 0.14 seconds for NSE, while 512 spectra for 216 patient records (110,592 spectra in total) were calculated in 55.79 ± 0.26 seconds for the radix-2 FFT implementation. Scaling by 32×, 3,538,944 spectra would be calculated in 1785.28 ± 8.32 seconds for FFT. This represents a speed advantage of 153:1 for NSE over FFT when the real-time analysis algorithm is implemented. Dividing each time by 3,538,944, the average time for a single real-time spectral calculation was 3.29 μs for NSE versus 504.5 μs for FFT. During a 1 millisecond sampling period, the NSE algorithm therefore has the capability to spectrally analyze a maximum of 303.95 data channels, although only 216 patient data sequences were actually analyzed for this study. By comparison, the FFT algorithm could only analyze a maximum of 1.98 channels, which for purposes of calculation means that the FFT can only analyze a single recording sequence in real time within the 1 millisecond sampling interval.

### Hardware implementation

**Figure 6:**
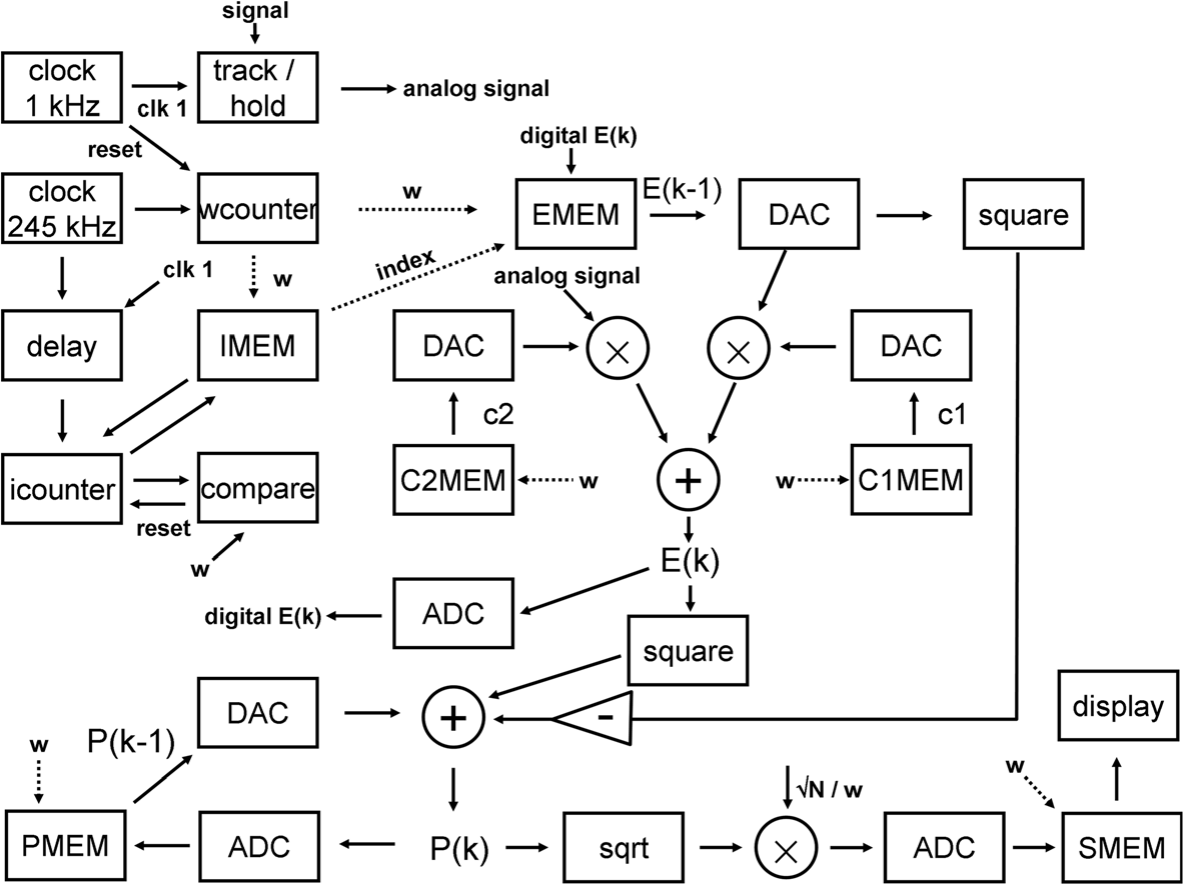
**Schematic block diagram of hardware implementation for the real-time NSE algorithm.** Solid arrows indicate data information while dotted arrows indicate addressing information. The addressing information is sent to memory chips (MEM). Memory chips require address selection from w = 81 to w = 325. The EMEM chip also requires addressing of the index pointer location for each w.

The schematic for hardware implementation based upon the NSE real-time software algorithm (spectral_estimate_real_time) is shown in Figure 6. The hardware consists of a mix of analog and digital components, and for simplicity it is shown as sampling the data at exactly 1.0 kHz (1 millisecond intervals) using a 1 kHz clock. To update parameters from w = 81-325 corresponding to a 12-3 Hz frequency range, which consists of 245 values, a 245 kHz clock was used. For ease of implementation, an off-the-shelf 250 kHz oscillator could just as readily be used. The data stream (signal) is tracked and then held with an edge of the 1 kHz clock (labeled clk 1). The analog signal is then valid (hold) until the next 1 kHz pulse. The edge of the 1 kHz clock waveform is also used to reset a counter (wcounter). This counter provides the current value of segment length w being analyzed, and it counts in increments of 1 from 81 to 325 on each pulse from the 245 kHz clock. The wcounter output (labeled w) is used as addressing information for the memory chips that are present on the electronics board, as well as for data input elsewhere in the circuitry. In the schematic diagram, addressing information is indicated by dotted lines, while data is noted by solid lines.

In Table 2, the characteristics of each memory chip on the board are shown. Stored in EMEM are the ensemble means for each segment length w from 81-325. Each ensemble mean is a vector of length w. Therefore the addressing information sent to the EMEM chip, when a newly calculated ensemble mean value is to be stored, must specify not only the segment length w (received from wcounter), but also the current index number *i* for that w that is being addressed (received as ‘index’ from the index memory chip–IMEM). Each time a new input sample point is received, the index number for each w is incremented by 1, with wraparound. As an example, for w = 100, the index begins at 1 and is increased to 2, 3, 4, …, 100 for each new input sample point (i.e., once every millisecond). When the index reaches 100 it must then wraparound to 1. This reset is done by use of the counter and comparator that are associated with IMEM. The index value contained in IMEM for segment length w is presented to the counter (icounter). Using a delayed pulse from the 245 kHz clock, the icounter is then incremented by 1. The icounter output after incrementation is compared to the current value of w (received from wcounter). If the icounter output is greater than w (i.e., its value is w + 1), it is reset to 1. The icounter output is then stored in IMEM at the address value w that is currently being accessed.

**Table 2:** Characteristics of the memory integrated circuits

**Name**	**Storage variable**	**Addressing**	**What is stored**	**Symbol**
C1MEM	Constant	245 values	Constant for MA equation	c1
C2MEM	Constant	245 values	Constant for MA equation	c2
EMEM	Ensemble mean	245 × w values	e.m. for each index at each w	*e*_*w*_
PMEM	Ensemble power	245 values	e.p. of each e.m.	*P*_*w*_
IMEM	Index	245 values	Points to an element of e.m.	*i*
SMEM	Spectrum	245 values	Spectral points	*S*_*w*_

w = segment length to compute the ensemble mean, 245 = the number of segment lengths for which the ensemble mean is calculated (from 81 to 325), e.m. = ensemble mean, e.p. = ensemble power, MA = moving average.

The digital ensemble mean value E(k) contained within EMEM for a particular w and index is output as E(k-1) to a digital-to-analog converter (DAC). The DAC output is multiplied by the constant c1. The new input sample point (analog signal in the diagram) is multiplied by the constant c2. The constants c1 and c2 are used to weight the moving average of the ensemble mean (line 16 of spectral_estimate_real_time), according to the following equation:

(19) $$E\left(k\right)=c1\times \mathrm{ensemble}\;\mathrm{mean}\;+\;c2\times \mathrm{input}$$

where, as computed in lines 6-7 of spectral_estimate_real_time:

(20) $$n\left(w\right)=\mathrm{int}\left(\frac{N}{w}\right)$$

with N being the window length, 8,192 sample points, ‘int’ is the integer function, and:

(21) $$c1\left(w\right)=\frac{n\left(w\right)-1}{n\left(w\right)}$$

(22) $$c2\left(w\right)=\frac{1}{n\left(w\right)}$$

Eq.’s 19-22 are analogous to Eq.’s 3 and 12-14 for the software implementation. The values of c1(w) and c2(w) are constant, and it is supposed that they can be stored in C1MEM and C2MEM prior to real-time spectral analysis, using a separate device to write to the chips, to maintain the standalone quality of the electronic board design. The two products on the right-hand-side in Eq. 19 are then summed. This is illustrated in Figure 6, with the result *E(k)* being input to an analog-to-digital converter (ADC). The resulting digital value of *E(k)* replaces *E(k-1)* in EMEM for the w and index currently being pointed to by the addressing information. The moving average filter output is also squared, which is then used as one of the elements inputted to a summing circuit to revise the value of the ensemble power P. The update of P at discrete time k is given by the equation:

(23) $$P\left(k\right)=P\left(k-1\right)+E{\left(k,-,1\right)}^{2}-E{\left(k\right)}^{2}$$

where Eq. 23 is analogous to Eq. 16 used for software calculation. The quantity *P(k-1)* is accessed from the ensemble power memory chip (PMEM). The quantity *E(k-1)*^*2*^ is obtained by squaring the output of the EMEM DAC. The output of this summing circuit is the analog power *P* at time *k*. This value is digitized using an ADC and stored as the new value of P at w in PMEM. The square root of *P(k)* is also obtained, which is then scaled according to Eq. 8c or 8d (for simplicity shown using Eq. 8c, √N / w), where √N = 90.51, and a DAC is used to convert w to analog form (for simplicity, not shown in Figure 6). The result is digitized and stored in the spectral memory (SMEM). SMEM contains 245 digital values representing the current spectral content from w = 81 to 325 (12-3 Hz). The SMEM digital output is then sent to a display. For simplicity, the spectral display device is not shown. To prevent the need to tie the spectral analyzer board to a computer, the output of SMEM could be sent, for example, to a dot matrix display with appropriate circuitry.In total according to Figure 6, approximately 26 integrated circuit chips, plus delay, display circuitry, and power supply (not shown) can be used to implement the real-time NSE algorithm on a prototyping electronics board. The circuitry must run at nearly 250 kHz; thus the settling time for the integrated circuits should be less than 4 microseconds, which necessitates the purchase of high quality components. The DAC and ADC integrated circuits should be parallel input and output, respectively, for faster throughput; thus single rather than dual or quad packages should be used for these devices. For reduced cost, an 8-bit digital resolution can be selected. For memory access, latching, and counting, sufficient delay will be required to allow inputs to each integrated circuit chip in Figure 6 to settle. This can be done for example using 555 timer circuits, and the associated resistors and capacitors, which for simplicity are not shown in the schematic.

Since the fast clock runs at approximately 250 kHz, the generation of each spectral point must be completed within four microseconds. As shown in Table 3, all components of Figure 6 except the display unit would be purchasable in high performance versions for approximately $500 at the current market pricing for individual components. The sample rate with real-time spectral update of the circuitry, outlined in Figure 6, could be improved to a maximum value of approximately 4 kHz, using a 1 MHz fast clock (this is the maximum run speed according to the longest settling times of 1 μs listed in Table 3). [μs is the microsecond symbol].

**Table 3:** Parts list

**Part**	**Company**	**Function**	**Settling time**	**Price**	**Quant**	**Total**
AD532	Analog devices	Analog mult, divide, square, sr	1 μs	$50	7	$350
SN74LS682	Texas instruments	CMOS comparator	30 ns	$2	1	$2
AS6C1008	Alliance memory	CMOS SRAM	55 ns	$2	6	$12
SI510	Silicon labs	CMOS output oscillator	1 ns	$50	2	$100
HA5351	Intersil	CMOS sample/hold	64 ns	$20	1	$20
MAX5595	Maxim integrated	CMOS DAC 8 bit parallel	1 μs	$5	5	$25
SN74HC393	Texas instruments	CMOS counter	100 ns	$1	2	$2
ADC08D1520	Texas instruments	CMOS ADC 8 bit parallel	1 μs	$5	3	$15

Mult = multiplication, sr = square root, ns = nanoseconds, μs = microseconds. Quantity of AD532 includes 3 multiplies, 2 squares, 1 divide by, 1 square root. The total estimated cost is $526 plus delay circuitry and any additional resistors and capacitors that are needed.

## Discussion

### Summary

In this study the NSE method was implemented for real-time analysis as a software algorithm and as a block diagram for a prototypical hardware electronics board. To be implemented in real time, the spectral estimate is updated within the time interval needed for the data stream to shift by the analysis window moving size of 1 sample point (M = 1), which for the application presented was 1 millisecond. The data stream consisted of sequences of retrospectively analyzed fractionated atrial electrograms from AF patients. The NSE algorithm was found to be implementable in real time using a few lines of software code. The mean time for calculation of one power spectrum was 3.29 μs for NSE versus 504.5 μs for the conventional radix-2 FFT implementation. Thus for real-time spectral analysis, the NSE algorithm was found to be over 150 × faster than the conventional FFT. Based on these values, for a 1 millisecond sampling period, the NSE algorithm has the potential to spectrally analyze over 300 data channels while the DFT algorithm can only analyze a single channel. Thus for NSE, although 216 sequences were actually analyzed, the number of sequences could be increased to 300 while maintaining real-time calculation within the 1 millisecond window moving size. Whereas for the conventional radix-2 FFT implementation, only one channel can be analyzed when the moving size M = 1. The rapid speed of NSE for real-time analysis is due to the low computational overhead in calculating the update as compared with the FFT recalculation.The NSE was found to be implementable in hardware using approximately 26 integrated circuits at a cost of approximately $500. No computer controller or digital signal processor would be needed to run the hardware implementation–it was developed as a standalone spectral analyzer board. Based on the settling times of the mixed-signal circuitry in Figure 6, the sampling rate could possibly be increased from 1 kHz to 4 kHz while maintaining a real-time spectral output. To analyze more channels simultaneously, the circuitry of Figure 6 would be repeated.

### Optimized implementations

The FFT implementation used for this study was not optimized, but was selected because the software code is in the public domain. To estimate the approximate speed advantage if an optimized FFT implementation were to be used, benchmark data can be compared for the Numerical Recipes implementation versus a ‘Fastest Fourier Transform in the West’ (FFTW) implementation termed ‘fftw3’ [21]. FFTW is a structured library of optimized C code blocks termed codelets, and is portable to any platform having a C compiler. It is used for computing the FFT in one or more dimensions, with any input size, for either real or complex data. Over a variety of platforms, FFTW’s performance has been shown to be improved over most other publicly available FFT software, and is competitive with commercial software [21]. Benchmark data was available from two CPUs with performance that is comparable to the CPU of the laptop computer used in this study–the 2 GHz PowerPC (970 (G5), 32 bit mode, gcc-3.4) and the 3.0 GHz Intel Core Duo (32-bit mode). For the Power PC implementation, benchmark data was available for computing the 1-dimensional FFT, single precision, with complex numbers. The Numerical Recipes in Fortran version, which was used in our study, was benchmarked at 600 megaflops, while the fftw3 version ran at 6000 megaflops. For the Intel Core Duo implementation, benchmark data was available to compute the 1-dimensional FFT, single precision, with real numbers. Numerical Recipes in Fortran was benchmarked at 1000 megaflops, while the fftw3 version ran at 8400 megaflops. Thus using a highly optimized FFT set of library functions and compiler, it is possible to obtain an approximate 10 × speed advantage as compared with the Numerical Recipes FFT implementation. Supposing that a 2 × speed advantage can be gained by including shortcuts to update the FFT in real time, it may therefore be possible to obtain overall as much as a 20 × speed increase in running a real-time FFT using optimized hardware and software as compared with the standard FFT used for our study. However, even if the best optimization protocols were implemented to speed up the FFT by approximately 20 ×, and no steps were taken to optimize the NSE algorithm, NSE would still be expected to run approximately 150/20 ~ 7.5 × faster than the FFT.

### Real-time spectral analysis

A comparison of four software algorithms for real-time spectral analysis of AF signals was recently studied [22]. The estimators used were the FFT, Blackman-Tukey (BT), Autoregressive (AR), and Multiple Signal Classification (MUSIC) methods. The window size was 4096 discrete sample points. Calculations were made for a number of segments and averaged. The investigators found the time for spectral calculation for 3000 signals. Scaling for 216 signals, time for calculation for FFT, BT, AR, and MUSIC was respectively, 1.6, 24.5, 14.9, and 781.3 seconds. Supposing the spectra of 10 segments were averaged, then the FFT computation time for one segment would be 0.16 seconds, which is comparable to our value for static implementation of 0.12 seconds. The study therefore suggests that the FFT is the fastest spectral estimator of the group, and that the runtime for the FFT is similar to that found in our study.

Hardware implementations of real-time spectral analyzers can be purchased either in the form of an electronics board which is inserted onto a computer backplane bus, or as a standalone mainframe unit. Electronic boards are less expensive to purchase but have limited portability because they require a dedicated computer for control of the board. Real-time standalone units can be quite expensive. Models such as the RSA5000 Spectrum Analyzer (Tektronix Inc., Beaverton, OR) can be used to view real-time spectra in the frequency range from DC to gigahertz, but the cost is $25,000 or more. The prototypical spectral analyzer described in our study, when sampling is at a projected maximum of approximately 4 kHz, would have a power spectrum range from DC to 2 kHz. It is implementable without the need for interfacing with a computer and would be a small, highly portable device. Although the bandwidth is limited, the range of frequencies is more than sufficient to analyze most electrophysiologic signals. Another use, for example, would be to display the frequency content of music or voice in real time.

If the real-time NSE is implemented in software it would be possible to improve the calculation time, for example, by using vectorized packages in Visual C++, such as with the OptiVec shareware package (OptiCode–Dr Martin Sander Software Dev) which was written for 64-bit platforms including Windows 7 and 8. The OptiVec is a set of assembler-written functions for both floating-point and integer data types that perform vectorized and matrix operations. As suggested by the documentation, runtime speeds can be improved by 2-3 ×. Use of such a program could therefore enable a substantial increase in the number of data channels that are spectrally analyzed by the NSE method in real-time at 1-4 kHz sampling, and/or to increase the sampling rate. Higher sampling rates will result in a greater temporal resolution, and can also be used to analyze higher frequency components, which could be useful for other applications.

### Clinical correlates

Analysis of multichannel electrogram data via spectral estimation has been shown useful to distinguish patients with paroxysmal versus persistent atrial fibrillation based upon frequency gradients [7]. Hence acquisition and real-time analysis of multichannel data is potentially assistive to characterize and localize the substrate changes that occur during atrial fibrillation. It may also be helpful to detect optimal ablation sites to prevent recurrence of arrhythmia [23,24]. In a prospective setting, rather than 216 different patient sequences as was done in this study, the data would be obtained from a multichannel electrode, for example using a noncontact [25] or basket [26] electrode. As the number of multichannel electrode recordings available in these devices is increased, the possibility of analysis of all of the channels in real time becomes more remote, yet would be necessary for optimally targeting arrhythmogenic regions for ablation. Thus, the implementation of a real-time spectral estimator for multichannel data is potentially important to improve clinical outcome.

The dominant frequency or DF is a useful parameter for catheter ablation of arrhythmogenic sites in AF patients [7]. When real-time spectral analysis is used to determine the DF during electrophysiologic mapping in patients with ongoing AF, it is possible to predict the long-term maintenance of sinus rhythm [27]. Real-time spectral analysis has also been used to show a strong correlation of the DF calculated from both unipolar and bipolar surface electrograms, to optical mapping data in ovine hearts [28]. The findings of that study suggest that estimation of activation rate via real-time spectral analysis of surface electrograms and DF calculation, correlates well with optical mapping measurement of the actual electrical activation rate. Thus, real-time spectral analysis of surface electrograms, as are acquired during clinical electrophysiologic study, provides physiologically relevant parameters of electrical activation that can be used for targeting arrhythmogenic regions.

Although our study was limited to bipolar electrogram analysis, the morphology of unipolar AF electrograms is reflective of specific patterns of conduction including broad fibrillation waves, collision of distinct wavelets, slow conduction, and pivoting fibrillation waves [29]. Thus unipolar recordings may be particularly useful to differentiate between AF types and to identify regions with different structural properties.

### Limitations

The NSE and DFT real-time algorithms were tested on retrospective data. Implementation on prospective multichannel data would be desirable to determine the speed of the spectral estimators in this setting. Still, in principle there should be no difference in speed by using 216 retrospective patient data sequences versus 216 prospective multichannel data sequences that are acquired simultaneously. A conventional FFT software algorithm rather than an optimized algorithm was used for testing. However, the conventional FFT is likely to be implemented for most analyses because of its simplicity and ready availability. The NSE and FFT algorithms were tested with one software compiler and one computer and operating system. Use of a different software compiler and computer may result in somewhat different computation times. For the hardware implementation, the design was illustrated only as a schematic block diagram. Actual implementation of the hardware design may necessitate changes to the components used.

## Conclusions

The NSE algorithm is implementable with low computational cost and complexity in both hardware and software. Real-time 1 millisecond updates of the spectral estimate can be done even when many tens or hundreds of data sequences are being acquired and analyzed during the same discrete time interval, as would be the case when using a multichannel basket electrode. The algorithm can be implemented as a standalone spectral analyzer board at a price of approximately $500 plus the cost of the display unit, without the need to interface with a computer. Although it is possible to improve the efficiency of the DFT real-time update and the compiler by as much as 20 × [1,2], such implementations would not match the improvement gained by using the NSE real-time algorithm, which was found by comparison of software algorithms to be approximately 150 × faster. The sampling rate using the hardware implementation of the NSE could possibly be further increased, being limited only by the settling times of the on-board components as compared with the fast clock speed. The realization of a fast spectral analysis algorithm is potentially helpful increase the number of multichannels analyzed, to characterize spectral transients in biomedical data as they occur, and to update in real time the detailed trends and gradients in the frequency content of biomedical data that may be present over longer sequences. This may be particularly helpful when probing the tissue substrate for anomalous regions, as is the case during electrophysiologic study of AF patients.

## Abbreviations

ADC: Analog-to-digital converter; AF: Atrial fibrillation; DA: Dominant amplitude; DAC: Digital-to-analog converter; DC: Direct current; DF: Dominant frequency; DFT: Discrete Fourier transform; FFT: Fast Fourier transform; FFTW: Fastest Fourier Transform in the West; MP: Mean spectral profile; NSE: New spectral estimator; SP: Standard deviation in spectral profile.

## Competing interests

The authors declare that they have no competing interests.

## Authors’ contributions

EJC did the quantitative work and wrote the manuscript. AB and HG acquired the clinical data, selected electrograms for analysis, and reviewed the manuscript. All authors read and approved the final manuscript.

## Supplementary Material

Additional file 1 Fortran.zip. Click here for file

Additional file 2 txtfiles.zip. Click here for file

## References

[B1] Bongiovanni G Corsini P Frosini G Procedures for computing the discrete Fourier transform on staggered blocks IEEE Transact Acoustics Speech Signal Process 1976 24 132 137 10.1109/TASSP.1976.1162787

[B2] Lo PC Lee YY Real-time implementation of the moving FFT algorithm Signal Process 1999 79 251 259 10.1016/S0165-1684(99)00098-5

[B3] Nibouche O Boussakta S Darnell M Benaissa M Algorithms and pipeline architectures for 2-D FFT and FFT-like transforms Digit Signal Process 2010 20 1072 1086 10.1016/j.dsp.2009.10.028

[B4] Huang HY Lee YY Lo PC A novel algorithm for computing the 2D split-vector-radix FFT Signal Process 2004 84 561 570 10.1016/j.sigpro.2003.11.018

[B5] Biviano AB Coromilas J Ciaccio EJ Whang W Hickey K Garan H Frequency domain and time complex analyses manifest low correlation and temporal variability when calculating activation rates in atrial fibrillation patients Pacing Clin Electrophysiol 2011 34 540 548 10.1111/j.1540-8159.2010.02993.x 21208232

[B6] Lin YJ Tai CT Kao T Tso HW Higa S Tsao HM Chang SL Hsieh MH Chen SA Frequency analysis in different types of paroxysmal atrial fibrillation J Am Coll Cardiol 2006 47 1401 1407 10.1016/j.jacc.2005.10.071 16580528

[B7] Sanders P Berenfeld O Hocini M Jaïs P Vaidyanathan R Hsu LF Garrigue S Takahashi Y Rotter M Sacher F Scavée C Ploutz-Snyder R Jalife J Haïssaguerre M Spectral analysis identifies sites of high-frequency activity maintaining atrial fibrillation in humans Circulation 2005 112 789 797 10.1161/CIRCULATIONAHA.104.517011 16061740

[B8] Ciaccio EJ Biviano AB Whang W Wit AL Garan H Coromilas J New methods for estimating local electrical activation rate during atrial fibrillation Heart Rhythm 2009 6 21 32 10.1016/j.hrthm.2008.10.016 19121796

[B9] Ciaccio EJ Biviano AB Whang W Coromilas J Garan H A new transform for the analysis of complex fractionated atrial electrograms BioMed Eng OnLine 2011 10 35 10.1186/1475-925X-10-35 21569421 PMC3125385

[B10] Ciaccio EJ Biviano AB Garan H Comparison of spectral estimators for characterizing fractionated atrial electrograms BioMed Eng OnLine 2013 12 72 10.1186/1475-925X-12-72 23855345 PMC3728006

[B11] Ciaccio EJ Biviano AB Garan H Computational method for high resolution spectral analysis of fractionated atrial electrograms Comput Biol Med 2013 43 1573 1582 10.1016/j.compbiomed.2013.07.033 24034749

[B12] Holm M Pehrson S Ingemansson M Sörnmo L Johansson R Sandhall L Sunemark M Smideberg B Olsson C Olsson SB Non-invasive assessment of the atrial cycle length during atrial fibrillation in man: introducing, validating and illustrating a new ECG method Cardiovasc Res 1998 38 69 81 10.1016/S0008-6363(97)00289-7 9683908

[B13] Pehrson S Holm M Meurling C Ingemansson M Smideberg B Sörnmo L Olsson SB Non-invasive assessment of magnitude and dispersion of atrial cycle length during chronic atrial fibrillation in man Eur Heart J 1998 19 1836 1844 10.1053/euhj.1998.1200 9886727

[B14] Jarman JW Wong T Kojodjojo P Spohr H Davies JE Roughton M Francis DP Kanagaratnam P Markides V Davies DW Peters NS Spatiotemporal behavior of high dominant frequency during paroxysmal and persistent atrial fibrillation in the human left atrium Circ Arrhythm Electrophysiol 2012 5 650 658 10.1161/CIRCEP.111.967992 22722660

[B15] Salinet JL Tuan JH Sandilands AJ Stafford PJ Schlindwein FS Ng GA Distinctive patterns of dominant frequency trajectory behavior in drug-refractory persistent atrial fibrillation J Cardiovasc Electrophysiol 2013 doi:10.1111/jce.12331 10.1111/jce.12331 24806529

[B16] Press WH Teukolsky SA Vetterling WT Flannery BP Numerical Recipes in Fortran 1992 NewYork: Cambridge University Press 501 502

[B17] Ciaccio EJ Biviano AB Whang W Gambhir A Garan H Spectral profiles of complex fractionated atrial electrograms are different in longstanding and acute onset atrial fibrillation atrial electrogram spectra J Cardiovasc Electrophysiol 2012 23 971 979 10.1111/j.1540-8167.2012.02349.x 22578068 PMC4287228

[B18] Everett TH IV Moorman JR Kok LC Akar JG Haines DE Assessment of global atrial fibrillation organization to optimize timing of atrial defibrillation Circulation 2001 103 2857 2861 10.1161/01.CIR.103.23.2857 11401945

[B19] Everett TH IV Verheule S Wilson EE Foreman S Olgin JE Left atrial dilatation resulting from chronic mitral regurgitation decreases spatiotemporal organization of atrial fibrillation in left atrium Am J Physiol Heart Circ Physiol 2004 286 H2452 H2460 10.1152/ajpheart.01032.2003 14962833

[B20] Mateo J Rieta JJ Radial basis function neural networks applied to efficient QRST cancellation in atrial fibrillation Comput Biol Med 2013 43 154 163 10.1016/j.compbiomed.2012.11.007 23228480

[B21] benchFFT http://www.fftw.org/benchfft/

[B22] Ahmad A Schlindwein FS Ng GA Comparison of computation time for estimation of dominant frequency of atrial electrograms: fast fourier transform, blackman tukey, autoregressive and multiple signal classification J Biomed Sci Eng 2010 3 843 847 10.4236/jbise.2010.39114

[B23] Nademanee K McKenzie J Kosar E Schwab M Sunsaneewitayakul B Vasavakul T Khunnawat C Ngarmukos T A new approach for catheter ablation of atrial fibrillation: mapping of the electrophysiologic substrate J Am Coll Cardiol 2004 43 2044 2053 10.1016/j.jacc.2003.12.054 15172410

[B24] Nademanee K Oketani N The role of complex fractionated atrial electrograms in atrial fibrillation ablation J Am Coll Cardiol 2009 53 790 791 10.1016/j.jacc.2008.11.022 19245971

[B25] Ciaccio EJ Chow AW Kaba RA Davies DW Segal OR Peters NS Detection of the diastolic pathway, circuit morphology, and inducibility of human postinfarction ventricular tachycardia from mapping in sinus rhythm Heart Rhythm 2008 5 981 991 10.1016/j.hrthm.2008.03.062 18598952 PMC2593141

[B26] Narayan SM Krummen DE Rappel WJ Clinical mapping approach to diagnose electrical rotors and focal impulse sources for human atrial fibrillation J Cardiovasc Electrophysiol 2012 23 447 454 10.1111/j.1540-8167.2012.02332.x 22537106 PMC3418865

[B27] Atienza F Almendral J Jalife J Zlochiver S Ploutz-Snyder R Torrecilla EG Arenal A Kalifa J Fernández-Avilés F Berenfeld O Real-time dominant frequency mapping and ablation of dominant frequency sites in atrial fibrillation with left-to-right frequency gradients predicts long-term maintenance of sinus rhythm Heart Rhythm 2009 6 33 40 10.1016/j.hrthm.2008.10.024 19121797 PMC2867332

[B28] Berenfeld O Ennis S Hwang E Hooven B Grzeda K Mironov S Yamazaki M Kalifa J Jalife J Time-and frequency-domain analyses of atrial fibrillation activation rate: the optical mapping reference Heart Rhythm 2011 8 1758 1765 10.1016/j.hrthm.2011.05.007 21699849 PMC3202688

[B29] Konings KT Smeets JL Penn OC Wellens HJ Allessie MA Configuration of unipolar atrial electrograms during electrically induced atrial fibrillation in humans Circulation 1997 95 1231 1241 10.1161/01.CIR.95.5.1231 9054854

